# Left Ventricular Hypertrabeculation: An Unexpected Cause of Stroke?

**DOI:** 10.7759/cureus.77572

**Published:** 2025-01-17

**Authors:** Sandra Cunha, Luís Luz, Mónica Amado, Bárbara Lemos, Behnam Moradi

**Affiliations:** 1 Internal Medicine, Unidade Local de Saúde da Região de Leiria, Leiria, PRT; 2 Cardiology, Unidade Local de Saúde da Região de Leiria, Leiria, PRT

**Keywords:** cardioemboólico, stroke, stroke in young population, ventricular hypertrophy, ventricular trabeculation

## Abstract

Stroke in young adults is considered a rare pathology, associated with a significant morbidity and mortality rate. In the etiological study of stroke in young people, it is important to carry out a comprehensive investigation as there is a greater propensity for undetermined causes, compared to the older population. Here, the authors present the clinical case of a 53-year-old male diagnosed with ischemic stroke, in which an extensive evaluation identified left ventricular hypertrabeculation as the presumed etiology of this event. This finding highlights the extreme importance of the etiological study, being particularly interesting due to the rarity of the findings.

## Introduction

Ischemic stroke in young adults is considered a rare pathology, with an incidence of 5-10%, with an increasing trend in recent years due to a greater sedentary lifestyle, increased consumption of alcohol and tobacco, as well as increased use of oral contraceptives [1-5]. Although the age limit is not fully defined, there are some studies that include adults up to 60-65 years old, with an increasing incidence with increasing age [1]. Regarding sex, there is a higher prevalence in females up to the age of 35, after which this difference diminishes and eventually disappears [2].

The importance of differentiating between strokes in young people and adults is linked to the etiological study carried out, which should be more comprehensive in the first group. In stroke in young people, it is important to exclude genetic, autoimmune, and infectious or non-infectious inflammatory causes. It is extremely important to carry out a comprehensive etiological study to exclude infectious (for example, syphilis or HIV) or non-infectious (for example, for example, systemic sclerosing lupus, anti-phospholipid antibody syndrome, and Takayasu arthritis) inflammatory causes, thrombophilia, and other genetic causes that affect cerebral blood circulation (for example, CADASIL (cerebral autosomal dominant arteriopathy with subcortical infarcts and leukoencephalopathy) disease) [2]. Thus, it is important to carry out studies on the main thrombophilia, anti-nuclear antibodies, and anti-neutrophil cytoplasmic antibodies, and exclusion of infections such as syphilis, HIV, and hepatitis.

In around one-third of cases, despite exhaustive investigation, stroke is considered cryptogenic [4]. Two retrospective studies carried out in two Portuguese hospitals show atherosclerosis as the main cause, followed by cardioembolic causes [1,3]. Noncompaction of the left ventricle is a rare cardiomyopathy, characterized by the presence of hypertrabeculation in the left ventricle, the prevalence of which is uncertain. Clinically, it can manifest itself in the form of ventricular arrhythmias, heart failure, and thromboembolic phenomena [6-8]. This report describes the case of a stroke patient with the possible etiology of left ventricular hypertrabeculation.

## Case presentation

A 52-year-old man presented to the emergency department with central facial paresis, right lower limb paresis, and hypoesthesia of the ipsilateral upper limb that had been evolving for 45 minutes. He was previously self-employed, ex-smoker (approximately eight pack-year), without regular medication, and had a family history of stroke at a young age. On physical examination at the time of hospital admission, the patient was normotensive and normocardic, with a sinus rhythm and unremarkable cardiopulmonary auscultation findings. Neurologically, the presenting symptoms were targetted and he scored 3 on the National Institutes of Health Stroke Scale (NIHSS). Analytically, there were no relevant changes, and head computed tomography (CT) with contrast showed left thalamocapsular hypodensity, with the absence of large vessel occlusion. He was started on double anti-platelet aggregation (acetylsalicylic acid and clopidogrel) and hospitalized to carry out an etiological study.

**Figure 1:**
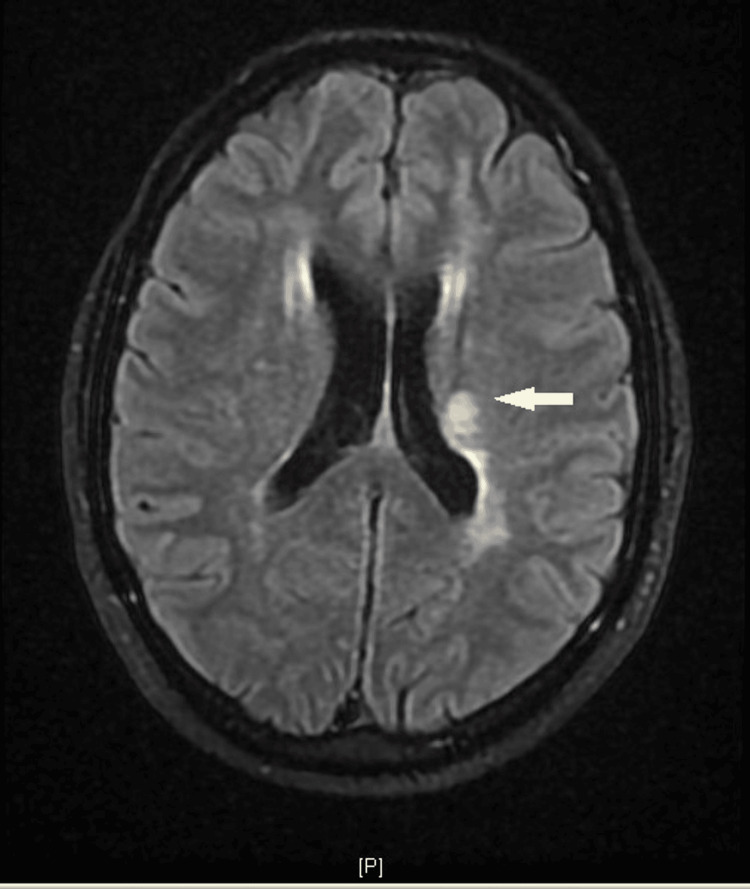
Brain MRI showing ischemic lesion in the left posterior lenticulocapsular region irrigated by the left thalamocapsular arteries

During hospitalization, he remained hemodynamically stable and there was complete neurological recovery. A cranial magnetic resonance imaging (MRI) was performed confirming an acute lesion in the left posterior lenticulocapsular region irrigated by the left thalamocapsular arteries, and scattered lesions were also described in the subcortical and peri-ventricular white matter with a predominance in the bilateral frontal regions, suggestive of chronic ischemic microvascular etiology (Figure 1).

**Figure 2:**
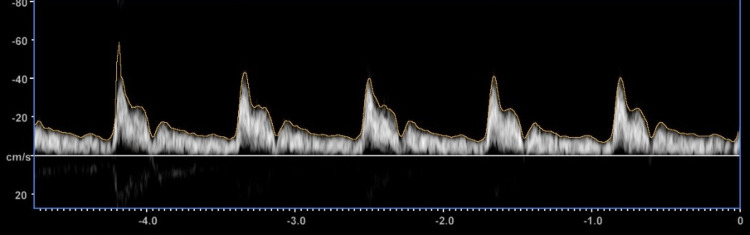
Cervical Doppler showing no relevant changes

**Figure 3:**
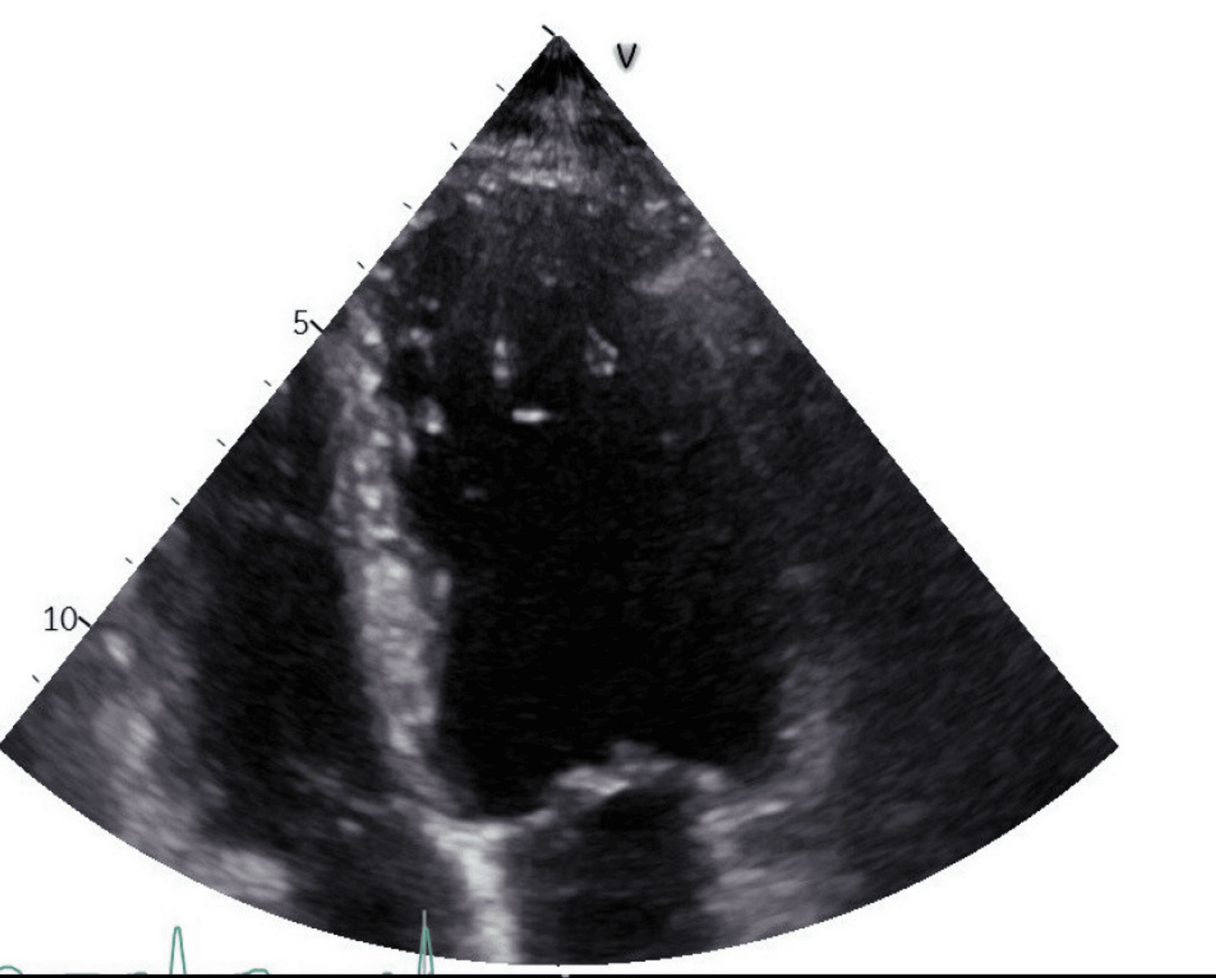
Transthoracic echocardiogram showing hypertrabeculation in the apical region of the left ventricle

In the analytical study (Table 1), there was homozygosity for the MTHFR A1298C variant, but with normal homocysteine levels, meaning there was no increase in thrombotic risk. A Doppler of the neck vessels and 24-hour Holter monitoring were performed without significant changes (Figure 2). To complete the study, a transthoracic echocardiogram was also performed, which documented a very trabeculated left ventricle in the apical region, with slight concentric hypertrophy of the ventricular walls and an ejection fraction at the lower limit of normal (~ 50% using the Simpson Biplane method) (Figure 3). 

**Table 1:** Laboratory investigations HDL: high-density lipoprotein; LDL: low-density lipoprotein

Exam	Values
Glycated Hemoglobin (HbA1c)	5.3%
Total Cholesterol	147 mg/dL
HDL cholesterol	36 mg/dL
LDL cholesterol	74 mg/dL
Total Cholesterol	210 mg/dL
Triglycerides	48 µmol/L
Uric Acid	Normal
Thyroid function	Normal
Sedimentation Velocity	4mm (Normal)
Protein Electrophoresis	Normal
Immunoglobulins (IgA, IgG and IgM)	Normal
Anti-nuclear antibodies	Negative
Anti-neutrophil cytoplasmic antibodies	Negative
Rheumatoid Factor	Negative
Protein C, S, and anti-thrombin III	Normal
Lupus anticoagulant	Negative
VDRL	Negative
HIV, hepatitis B and C serology	Negative
Anti-Beta2 glycoprotein, Anti-cardiolipin antibodies (IgG and IgM)	Negative
Antibodies (IgG and IgM)	Negative
*MTHFR A1298C* mutation	Homozygosity
Factor II and V	Normal

The case was discussed with Cardiology which, given the increased thrombotic risk, recommended initiating anticoagulation (although cardioembolic complications such as stroke, may occur infrequently). The patient was referred to this specialty for an MRI, which remains pending as of this date. Thus, in this clinical case, the non-compaction of the left ventricle was assumed to be a possible etiological cause and an oral anticoagulant was used to prevent cardioembolic events. A high-intensity statin was also started to obtain a low-density lipoprotein (LDL) cholesterol value below 55 mg/dL.

## Discussion

Stroke is the second most common cause of death in the world and the third cause of morbidity in the world [9]. In stroke at a young age, there tends to be a better prognosis, but this also has an important contribution to morbidity and mortality [4]. The etiological workup tends to be more extensive in young stroke patients compared to those of older age. However, in approximately one-third of cases, the cause remains cryptogenic, with atherosclerotic and cardioembolic causes being the most common [1,4,5].

In the current case, an exhaustive study was carried out to evaluate the main cardiovascular risk factors (hypertension, diabetes mellitus, dyslipidemia, overweight/obesity, and hyperuricemia), exclusion of atherosclerosis, sources of embolism, infectious, inflammatory, and autoimmune causes. The laboratory test results showed a slight increase in LDL cholesterol (with initiation of high-intensity statins to obtain a target of 55 mg/dL, since this is a patient with very high cardiovascular risk) and a mutation homozygous in the MTHFR A1298C gene, which only increases thrombotic risk if associated with an increase in homocysteine. The imaging tests performed revealed hypertrabeculation of the left ventricle on the transthoracic echocardiogram, and its contribution to the occurrence of this cerebral event was presumed, due to the increased embolic risk.

Analyzing the available literature, it appears that stroke can be a form of presentation of non-compaction of the left ventricle [6,8]. It should be noted that, at the time of writing this report, the cardiac resonance that confirms the diagnosis with a relationship between the thickness of the non-compact and compact layer > 2.3 [8] had not yet been performed; therefore, this etiological diagnosis is, to date, just very likely but not confirmed.

## Conclusions

From the etiological study carried out in this case, homozygosity for the MTHFR A1298C variant as well as hypertrabeculation of the left ventricle can be considered probable causes. However, as homocysteine ​​levels are normal, the latter was presumed to be the cause. However, the confirmatory MRI is still pending.

There is an increasing prevalence of stroke in young people, largely due to lifestyle changes that contribute to a rise in cardiovascular risk factors, a trend not observed in this clinical case. In most cases, stroke at a young age is not caused by traditional risk factors, and it is extremely important to carry out a comprehensive etiological study.
